# A transfer learning framework to elucidate the clinical relevance of altered proximal tubule cell states in kidney disease

**DOI:** 10.1016/j.isci.2024.109271

**Published:** 2024-02-22

**Authors:** David Legouis, Anna Rinaldi, Daniele Malpetti, Gregoire Arnoux, Thomas Verissimo, Anna Faivre, Francesca Mangili, Andrea Rinaldi, Lorenzo Ruinelli, Jerome Pugin, Solange Moll, Luca Clivio, Marco Bolis, Sophie de Seigneux, Laura Azzimonti, Pietro E. Cippà

**Affiliations:** 1 Division of Intensive Care, Department of Acute Medicine, University Hospital of Geneva, 1205 Geneva, Switzerland; 2 Laboratory of Nephrology, Department of Medicine and Cell Physiology, University Hospital and University of Geneva, 1205 Geneva, Switzerland; 3 Laboratories for Translational Research, Ente Ospedaliero Cantonale, Bellinzona, Switzerland; 4 Division of Nephrology, Department of Medicine, Ente Ospedaliero Cantonale, 6900 Lugano, Switzerland; 5 Istituto Dalle Molle di Studi sull'Intelligenza Artificiale (IDSIA), USI/SUPSI, Lugano, Switzerland; 6 Division of Pathology, Department of Diagnostic, University Hospital of Geneva, 1205 Geneva, Switzerland; 7 Division of Nephrology, Department of Medicine, University Hospital of Geneva, 1205 Geneva, Switzerland; 8 Institute of Oncological Research, 6500 Bellinzona, Switzerland; 9 Ente Ospedaliero Cantonale, 6900 Lugano, Switzerland; 10 Institute of Oncology Research, Università della Svizzera Italiana, Bellinzona, Switzerland; 11 Laboratory of Computational Oncology, Department of Oncology, Istituto di Ricerche Farmacologiche Mario Negri, Milano, Italy; 12 Faculty of Biomedical Sciences, Università della Svizzera Italiana, 6900 Lugano, Switzerland

**Keywords:** Cell biology, Integrative aspects of cell biology, Transcriptomics, Machine learning

## Abstract

The application of single-cell technologies in clinical nephrology remains elusive. We generated an atlas of transcriptionally defined cell types and cell states of human kidney disease by integrating single-cell signatures reported in the literature with newly generated signatures obtained from 5 patients with acute kidney injury. We used this information to develop kidney-specific cell-level information ExtractoR (K-CLIER), a transfer learning approach specifically tailored to evaluate the role of cell types/states on bulk RNAseq data. We validated the K-CLIER as a reliable computational framework to obtain a dimensionality reduction and to link clinical data with single-cell signatures. By applying K-CLIER on cohorts of patients with different kidney diseases, we identified the most relevant cell types associated with fibrosis and disease progression. This analysis highlighted the central role of altered proximal tubule cells in chronic kidney disease. Our study introduces a new strategy to exploit the power of single-cell technologies toward clinical applications.

## Highlights

• Single-cell atlas of transcriptionally defined cell types/states of kidney disease
• A transfer learning model for dimensionality reduction applicable to small datasets
• Computational model to link clinical data to kidney cell states using bulk RNAseq
• Evidence for a central role of altered proximal tubule cells in kidney fibrosis

## Graphical abstract

**Figure:**
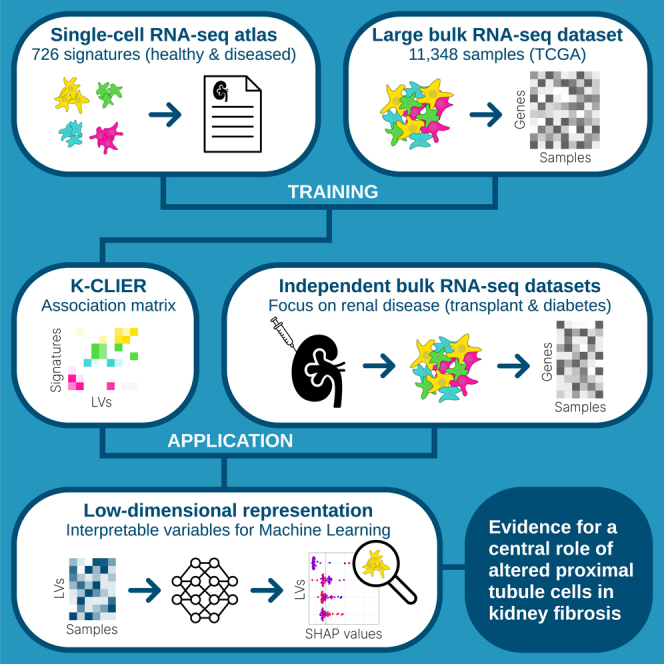

## Introduction

Translating the power of single-cell technologies into clinical research is predicted to boost the development of precision medicine by redefining diagnostic and prognostic tools and by guiding new discoveries in biomedical science. However, the applicability of single-cell technologies in large clinical studies or in clinical routine is limited by technical issues related to sample processing, costs, and data analyses.^1^^,^^2^ Single-cell technologies are particularly promising to study clinical conditions in organs with a complex cellular architecture. Kidney disease is a paradigmatic example: the kidney is built by > 30 different cell types, precisely arranged in complex anatomical structures. Chronic kidney disease (CKD) is a heterogeneous clinical condition, defined as a persistent abnormality in kidney structure or function for more than 3 months. CKD affects ca. 10% of the global population and is associated with adverse clinical outcomes, including end-stage kidney disease and death.^3^ Recent studies in experimental models and in small numbers of patients applied single-cell RNAseq (scRNAseq) or single-nucleus RNAseq (snRNAseq) to identify altered cell states associated with CKD.^4^^,^^5^^,^^6^^,^^7^^,^^8^^,^^9^^,^^10^ Evidence from experimental models suggests that altered tubule cells interact with surrounding cells to form cell communities involved in chronic inflammation and fibrosis, eventually leading to irreversible damage and CKD progression.^11^^,^^12^^,^^13^^,^^14^ The relevance of those findings needs to be validated in patients.^14^

Mao et al. developed a generally applicable unsupervised transfer learning method to extract information related to biological pathways from bulk RNAseq, the pathway-level information extractor (PLIER).^15^ The PLIER framework consists of two phases: the training phase applies a matrix decomposition method driven by prior knowledge (expressed in the form of gene signatures) to large publicly available RNAseq data to learn patterns of correlated genes, named latent variables (LVs), allowing a low dimensional representation of gene expression. In the second phase, the PLIER is applied to bulk RNAseq data from clinical tissue samples to obtain LV values of each sample. LVs can be handled as new features and be correlated to clinical data of interest. This approach was exploited by Taroni et al. by using large public data compendia to train the models, which could then be transferred to smaller datasets, expanding its applicability to rare diseases or clinical settings with limited numbers of patients (MultiPLIER).^16^

We developed a computational framework inspired by the MultiPLIER to translate the knowledge on kidney biology obtained by single-cell technologies in limited number of patients to a broad clinical context. Our framework specializes the MultiPLIER concept to kidney-specific cell types and states. We first constructed a single-cell reference atlas of human kidney disease. We trained and validated this kidney-specific cell-level information extractor (K-CLIER) and we applied it to bulk RNAseq datasets obtained from kidney biopsies in patients with kidney disease. Cell states associated with chronic kidney injury and progression to fibrosis were further characterized in a newly generated snRNAseq dataset obtained in patients in the early phase after acute kidney injury (AKI). Our integrated approach highlighted the central role of altered proximal tubule (PT) cells in the progression of chronic CKD in humans and introduces a new paradigm to translate single-cell technologies toward clinical applications.

## Results

### Atlas of transcriptionally defined cell states in human kidney disease

**Figure 1:**
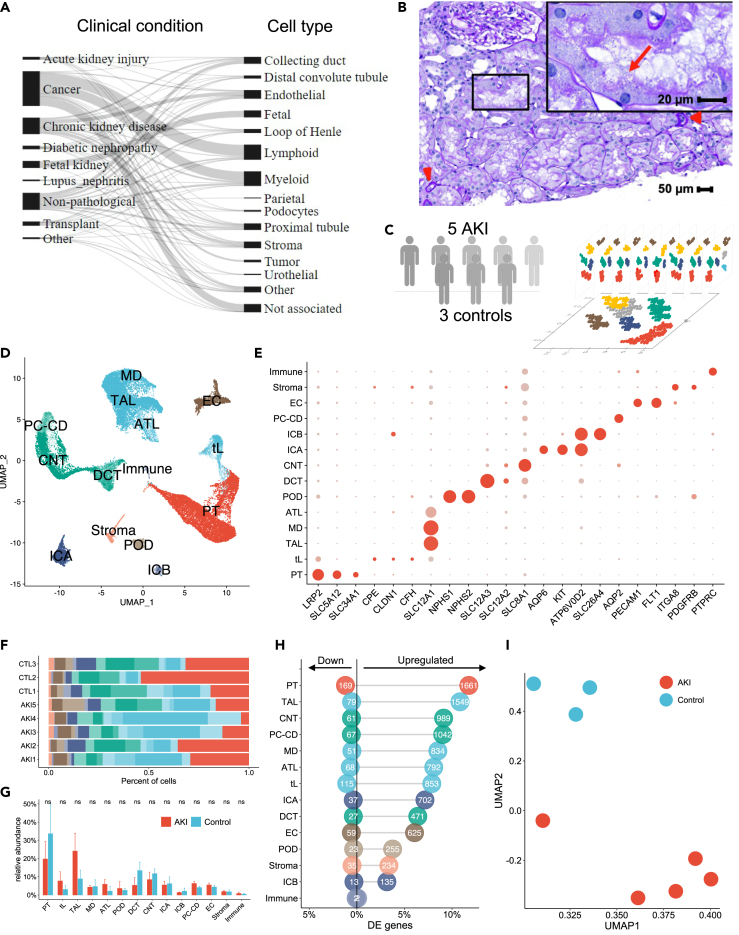
Atlas of human transcriptional profiles of kidney cells (A) Overview of the atlas composition. (B) Histopathology of a representative renal cortical area showing diffuse acute tubular lesions characterized by nonisometric sloughing of tubular epithelial cells and brush border loss (boxed area). Sparse foci of atrophic tubules are noted (red arrows heads). Red arrow indicates complete loss of the brush border. Periodic Acid-Schiff stain. (C) Schematic of the data integration strategy. (D) Uniform manifold approximation and projection (UMAP) representation of the 13 cell types identified by unsupervised clustering of the whole dataset. (E) Dotplot of cell cluster marker genes identified in AKI and control samples (dot size indicates the percentage of positive cells and color indicated relative expression). (F) Relative abundance of major cell types in each sample. (G) Relative abundance of major cell types in individuals with AKI and controls (mean with bootstraped confidence interval). (H) Absolute and relative numbers of differentially expressed genes upregulated and downregulated in AKI versus controls within major kidney cell types. (I) Principal component analysis of all study individuals using pseudobulk data per individual from all proximal tubule (PT) cells. See also Table S1.

To translate information from single-cell transcriptomics to clinical dataset, we first generated an atlas of transcriptionally defined cell types and cell states of the human kidney. We systematically included all renal single-cell signatures reported in the literature obtained by scRNAseq or snRNAseq in human kidneys up to March 2022. The cell signatures were classified according to clinical condition and cell type to facilitate the general interpretation of the results (Figure 1A). The available signatures covered all cell types of the normal kidney and several altered cell states reported in the most relevant subacute/chronic kidney diseases (including cancer and kidney transplantation), but there was a lack of data related to AKI (AKI). This was not surprising, since kidney biopsies are rarely performed in severely ill patients with AKI, but were considered a potentially important limitation for the next steps of the study in consideration of the peculiar cell states identified in experimental models in the early phase of AKI.^9^^,^^10^ Therefore, we expanded the cell-signature atlas by performing snRNAseq analysis on kidney biopsies obtained in 5 critically ill patients with COVID-19 associated AKI in the context of cytokine storm and multi-organ failure before planned withdrawal of resuscitation measures. The 5 patients suffered from stage 1–3 AKI, as defined by the KDIGO classification in consideration of serum creatinine or urine output, and kidney biopsies were obtained 6 to 26 days after AKI diagnosis. All patients displayed tubular alterations at different stages after tubular injury and of variable severity (Figure 1B). The clinical characteristics of the patients are presented in Table 1.

**Table 1:** Clinical characteristics of the patients

	Normal range	#1	#2	#3	#4	#5
**Demographics**						

Gender		female	male	female	male	male
Age		79	57	69	76	69
BMI (kg/m2)		34	25	20	28	26
Race		Caucasian	Caucasian	Caucasian	Caucasian	Caucasian

**Comorbidities**						

Hypertension		1	0	0	1	0
Diabetes		1	0	0	0	0
Chronic obstructive pulmonary disease		0	0	0	0	0
Chronic kidney disease		0	0	0	0	0
History of cancer		1	1	0	0	0

**Respiratory involvement, complications and severity scores**						

Mechanical ventilation (days)		10	2	21	17	26
VV-ECMO		0	0	0	0	1
Septic shock		0	1	0	0	1
Deep vein thrombosis		0	0	0	0	1
APACHE II score		19	33	8	30	15
SAPS II score		82	78	40	70	46
SOFA score		7	8	7	3	9

**Lab values at the day of kidney biopsy**						

Hemoglobin (g/L)	140–180	80	67	74	117	49
Leukocytes (G/L)	4–11	9.3	12	10.4	8.5	24.9
Platelets (G/L)	150–350	326	13	191	262	166
Neutrophils (G/L)	1.5–7.5	8.18 (88%)	10.08 (84%)	9.05 (87%)	7.57 (89%)	22.41 (90%)
Lymphocytes (G/L)	1–4.5	0.09 (1%)	0.36 (3%)	0.94 (9%)	0.34 (4%)	0.87 (3.5%)
D-Dimer (ng/mL)	45–500	5416	8810	795	2459	9865
LDH (U/L)	87–210	339	463	360	257	572
C3 (g/L)	0.66–1.35	1.75	1.48	1.3	1.78	0.8
C4 (g/L)	0.08–0.34	0.28	0.26	0.19	0.27	0.2
TNFa (pg/mL)	<4	17.96	5.6	6.16	1.42	3.2
IL6 (pg/mL)	<1.5	73.9	1190.5	474.1	3.76	6.8
IL8 (pg/mL)	<90	122.5	84.2	290	45.91	32.5
IL10 (pg/mL)	<1	2.5	5.9	3.33	<1	3.8
MCP1 (pg/mL)	50–260	1539	1243.2	1715	302	115.2

**Kidney function**						

Baseline serum creatinine (μmol/L)		150	95	66	76	80
Peak serum creatinine (μmol/L)		351	586	80	96	109
Serum creatinine at sampling (μmol/L)		175	219	61	62	39
AKI stage (KDIGO)		2	3	2	2	1
AKI criterium		creatinine	creatinine	oliguria	oliguria	oliguria
Days between AKI onset and biopsy		6	9	18	15	25
Renal replacement therapy		0	1	0	0	0

**Urinalysis at the day of biopsy**						

Protein (g/L)		0.56	1.21	1.26	0.44	0.13
Albumin (mg/L) (<10)		65	82	42	12	11
Creatinine (mmol/L)		1.9	2.3	3.9	5.3	3.3
Phopshate (mmol/L)		10.5	18.4	23	50	18.2
Magnesium (mmol/L)		2.33	2.41	5.18	6.37	2.22

**Histological findings**						

Tubule		Severe tubular atrophy	Moderate acute tubular lesion and atrophy	Moderate acute tubular lesion	Slight tubular atrophy	Moderate tubular atrophy
Interstitial fibrosis		Slight	Slight	Slight	Moderate	Slight
Interstitial inflammation		Focal	Absent	Focal	Absent	Absent
Pathology others			TMA		HN	

AKI: acute kidney injury; HN: hypertensive nephropathy; TMA: thrombotic microangiopathy; VV-ECMO veno-venous extracorporeal membrane oxygenation.

To verify the cluster annotation and to identify cell types, the new data were integrated with 3 controls from available datasets generated with similar tissue processing and single-cell technology (Figure 1C).^17^ Based on established cell type markers we identified all expected kidney cell types (Figures 1D and 1E). There were no major differences in major renal cell type abundances between AKI and control samples (Figures 1F and 1G). To assess the cell type-specific transcriptional response to AKI, we used a pseudo-bulk approach and performed differential gene expression analysis within the major kidney cell types comparing AKI to control kidneys through EdgeR. Profound transcriptomic responses to AKI, were observed in kidney tubule cells of the loop of Henle (including the thin limb—tL—and the thick ascending limb—TAL), the principal cells of the collecting duct (PC-CD) and the connecting tubule (CNT), and were maximal for the PT cells (Figure 1H). Consistently, dimensional reduction by UMAP shows a clear separation between AKI and control samples (Figure 1I). Therefore, the dataset was considered appropriate to define cell signatures of AKI, particularly to characterize injured tubule cells in the early phase of human AKI. After integration of the newly generated cell signatures of AKI, the atlas included 726 cell signatures (Figure 1A; Table S1).

### Training and validation of the K-CLIER framework

**Figure 2:**
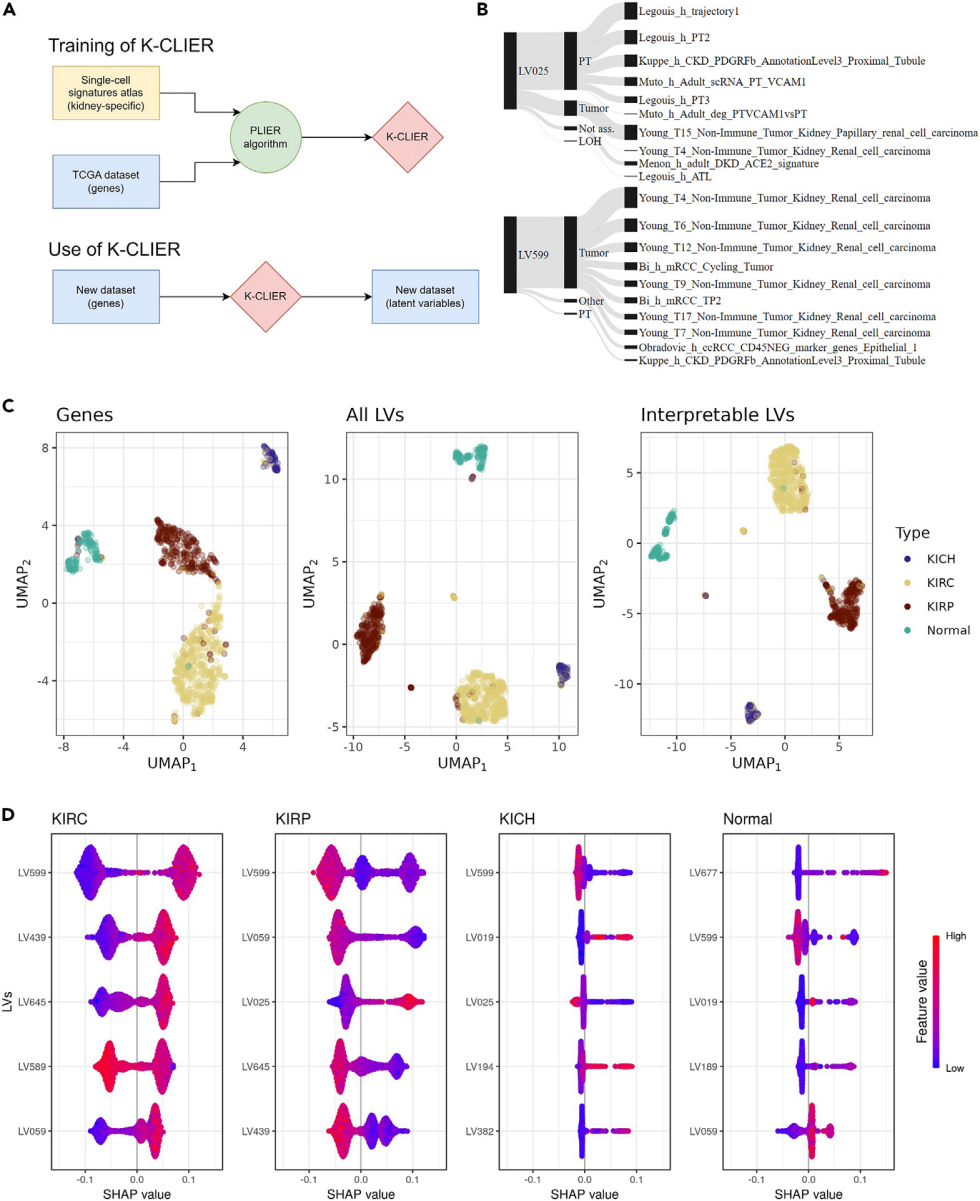
Validation of K-CLIER framework (A) Schematic representation of K-CLIER framework. (B) Association between latent variable (LV), cell type and single-cell signature for two relevant LVs. (C) Graphical two-dimensional representation of the original KIPAN gene expression dataset (31k genes), the dataset obtained by applying K-CLIER to it (700 LVs), and a subset of this latter containing interpretable LVs (96). UMAP was applied to all datasets. Samples are colored according to their histological type. (D) Most characterizing LVs for the different histological types in KIPAN dataset. Characterization is provided by training a model for prediction of the histological type and extracting Shapley values (the five most relevant LVs are shown). For each histological type, LVs with a positive impact on the target show positive Shapley values for higher-value of the LV (in red), e.g., LV599 for KIRC, whereas LVs with a negative impact show positive Shapley values for lower-value of the LV (in blue), e.g., LV599 for KIRP. See also Table S2.

The K-CLIER framework was trained by using 11.348 bulk RNAseq transcriptomes from 33 cancer types obtained from the cancer genome atlas program (TCGA) and the scRNAseq signatures of our kidney cell atlas as prior knowledge to drive the identification of latent variables (LVs) associated with specific cell types or cell states (Figure 2A). This reduced the number of variables for each sample from 18.630 genes to 700 LVs. Among the 700 LVs generated by the K-CLIER 96 were interpretable, i.e., strongly associated to cell types/states. Some LVs were associated with an individual single-cell signature (e.g., LV406 was associated with a specific type of fibroblasts, identified by Kuppe et al.^4^), others were associated with multiple signatures of similar cells identified by different groups (e.g., LV182 was associated with plasma cell signatures characterized in different settings), others were associated with different cell types/states (e.g., LV025, see Figure 2B). The association between the LV, the cell type and the specific scRNAseq signatures is reported in Table S2 and two relevant examples of LV associated with different cell types/states are shown in Figure 2B.

We validated the K-CLIER by testing its capability to retain information about the histological cancer type while reducing the data dimensionality. The TCGA dataset includes bulk RNAseq data of 1.026 kidney tumors (KIPAN dataset), including clear cell carcinoma (KIRC), papillary cell carcinoma (KIRP), chromophobe carcinoma (KICH), and normal samples. We applied the K-CLIER to calculate the LVs of such samples; then we applied UMAP to visualize in a two-dimensional space (i) the original gene expression profiles, (ii) the 700 calculated LV, and (iii) the subset of 96 interpretable LVs. In all cases we obtained 4 distinct clusters reflecting the histological cancer types (Figure 2C), indicating that the information was properly retained by the K-CLIER dimensionality reduction. For a quantitative evaluation, we trained a random forest classification model in a cross-validation setting using the 700-dimensional LVs dataset or the 96 interpretable LVs. In both cases we obtained an almost-perfect classifier (AUROC 0.99), thus confirming that histological type information is retained through the K-CLIER transformation. Next, we applied SHAP^18^ to a random forest model trained on the interpretable-LV dataset to quantify the contribution of each LV to the prediction for a given histological type and we extracted the LVs most associated with each histological type (Figure 2D). LV599, associated with different single cell signatures of clear cell carcinoma (Figure 2B), was most strongly associated with the histological type KIRC (Figure 2D). KIRP was negatively associated with LV599 and positively with LV025, which was related to a papillary renal cell carcinoma signature identified by Young et al.^19^ and to injured PT cells (further characterized in the following section). KICH was not directly associated to specific tumor cell types, but with LVs related to the collecting duct and more specifically with intercalated cells (LV19) according to the postulated cellular origin of this cancer type.^20^ Thus, the application of the K-CLIER to bulk RNAseq data not only allows a substantial dimensionality reduction without loss of relevant information, but—through the association between LVs and the single-cell signatures—also introduces the possibility to link clinical information to individual cell types or cell states included in our cell atlas of human kidney disease.

### Cell types associated with chronic kidney disease and fibrosis

**Figure 3:**
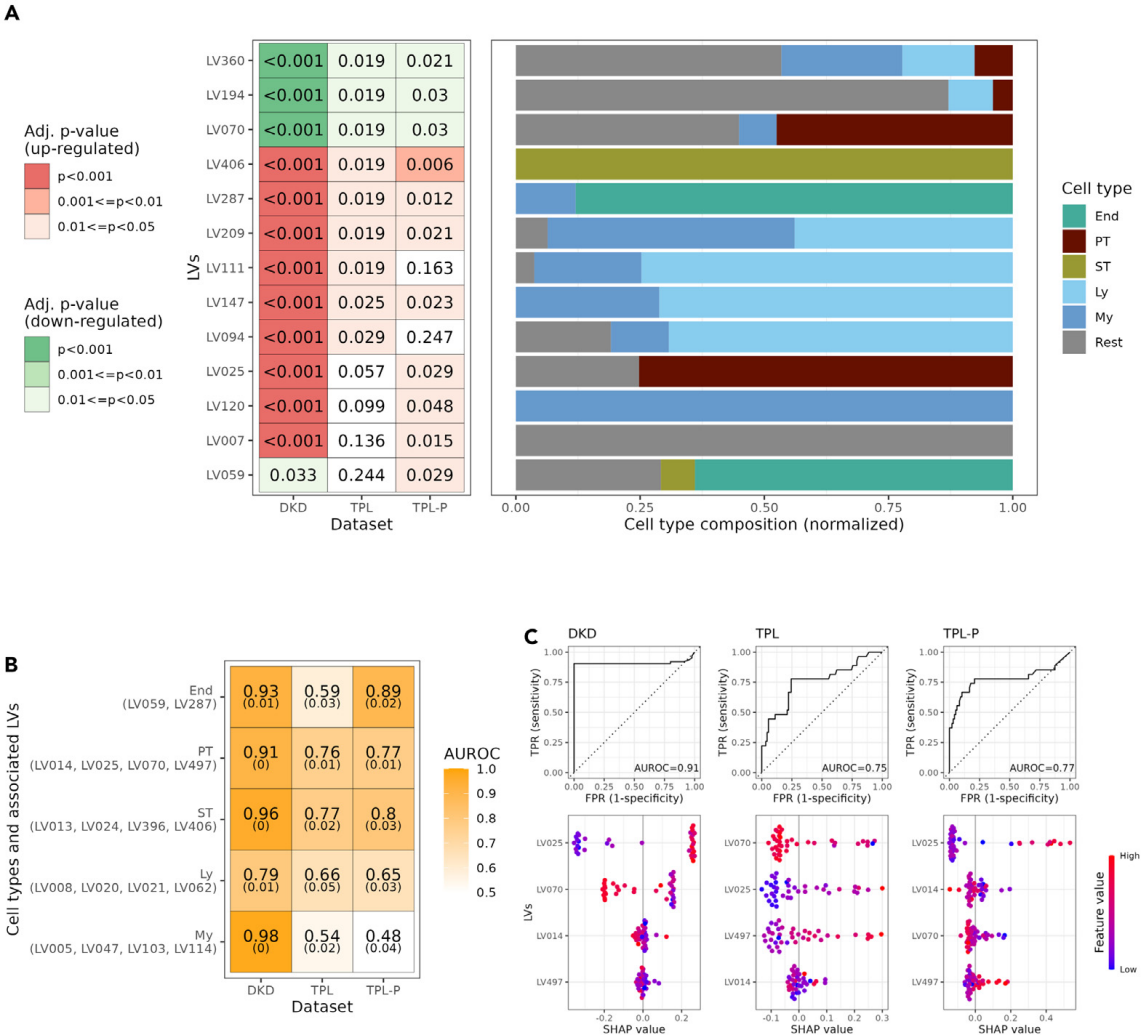
K-CLIER for studying CKD (A) Most significant interpretable LVs for distinguishing patients with and without CKD in DKD, TPL and TPL-P cohorts. The set of LVs with adjusted p value <0.05 both in the DKD and in at least one of the TPL cohorts is shown. Hypothesis tests were limited to interpretable LVs. End, endothelial; PT, proximal tubule; ST, stroma; Ly, lymphoid; My, Myeloid; Rest includes all other cell types (tumor, loop of Henle, distal convolute tubule collecting duct, podocytes, urothelial, fetal, parietal, not associated, other). (B) CKD predictive power of cell-type-specific sets of LVs. For each cell type, its 4 (if present) most associated LVs are extracted. These are used to train models for prediction of CKD in the DKD, TPL and TPL-P case (in 10-fold 10-repetition cross-validation). AUROCs of such models and their standard deviations are shown. (C) Focus on the models to predict CKD in DKD, TPL, and TPL-P cohorts using PT LVs. In the upper panel, ROC curves from models trained in cross validation; in the lower panel, Shapley values from models trained on the entire datasets. See also Table S2.

We identified cell states associated to CKD by applying the K-CLIER to bulk RNAseq data in kidney biopsies from two cohorts of patients. The first cohort (DKD) consisted of 28 patients with diabetic kidney disease and 9 controls.^21^ The second cohort (TPL) included 42 kidney transplant recipients, who underwent protocol biopsies 3 and 12 months after transplantation.^22^^,^^23^ Patients were classified according to the histological evaluation reported by the authors: in the DKD cohort, chronic kidney injury was defined as histological evidence of advanced nephropathy (in contrast to controls and early nephropathy), in the TPL cohort was defined according to the ci-score in the Banff classification (ci-score>1) evaluated 12 months after transplantation. Among the 96 interpretable LVs, we determined the LVs significantly associated to chronic kidney injury in both cohorts and we further considered only LVs displaying a significant association in both cohorts (Figure 3A). As expected, we obtained a higher degree of significant LVs in the DKD cohort, reflecting a more advanced nephropathy in these patients. The LVs negatively associated to chronic kidney injury were all associated to cells PT (LV070, LV194, and LV360), whereas positively associated LVs were mainly related to lymphocytes (Ly) and myeloid cells (My) (LV209, LV147, LV287, LV111, and LV094). LV406, associated to stroma cells, displayed a strong association with fibrosis in both cohorts. Thus, the K-CLIER correctly identified the transcriptional signature of PT cells as a characterizing cell type in uninjured kidney biopsies, and highlighted inflammation and stroma as the main characteristics of chronic kidney injury. Next, we evaluated if specific cell types were associated not only with established damage but also with progression to fibrosis. We took advantage of the fact that in the transplant cohort we could correlate RNAseq data (and therefore the LV obtained by applying the K-CLIER) from protocol biopsies at 3 months with the histological evaluation for fibrosis in the same kidney at 12 months after transplantation. Some of the LVs most strongly associated to progression to fibrosis were LV406, associated to a fibroblast population (p = 0.006); LV287, associated to endothelial cells (p = 0.012); LV007, associated to tubular and urothelial epithelial cells (p = 0.015).

To specifically address the potential role of individual cell types in CKD, we extracted a subset of LVs strongly related to each major kidney cell type and we trained models for predicting established fibrosis (DKD, TPL) or progression to fibrosis (TPL-P). The performance of each cell type specific model was quantified by the area under the ROC curve (AUROC), which was used as a measure of association between cell types and outcomes (Figure 3B). PT and stroma cells were associated to both established and progression to fibrosis. In the transplant cohort lymphocytes were weakly associated to the adverse outcome, whereas we did not find a significant association with myeloid cells, and endothelial cells showed a strong association only with progression to fibrosis. Notably, within the PT compartment some LVs were associated with a favorable and some with a negative outcome, suggesting a potential role of different cell states in PT states in the progression to fibrosis. Therefore, we applied SHAP to quantify the contribution of individual PT-associated LVs to fibrosis (Figure 3C). LV025 was positively associated with fibrosis in all three datasets, whereas LV070 showed a negative association in DKD and TPL. LV070 was mostly related to normal PT cells, as characterized by different authors (Table S2). LV025 included cell signatures of PT cells marked by vascular cell adhesion molecule 1 (VCAM1), identified as injured PT cells by Muto et al.,^6^ and reminiscent of late injured cell states previously characterized in mice after ischemia-reperfusion injury.^9^^,^^10^ Notably, LV025 was also associated to the set of genes discriminating the VCAM1 positive cells from other PT cells in the same dataset, highlighting the specific features of this subpopulation. LV025 was also associated with PT cells identified in the newly generated single cell dataset of human AKI. Thus, through a transfer learning approach we found a strong association between cell signatures of altered PT cell states and kidney fibrosis in patient biopsies.

### Characterization of altered proximal tubule cells

**Figure 4:**
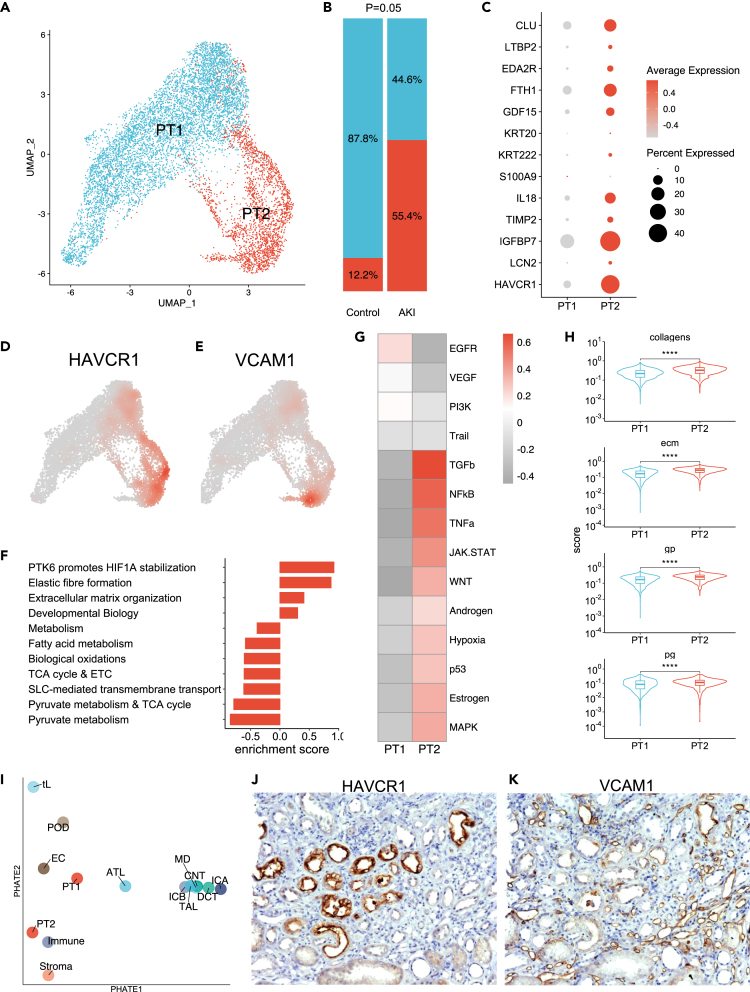
Characterization of altered proximal tubule cells (A) UMAP representation of the PT compartment from AKI and control patients. (B) Relative abundance of PT1 and PT2 clusters in AKI and control patients. (C) Expression of known injury marker genes in PT1 and PT2 clusters. (D and E) Gene-weighted density of HAVCR1 and VCAM1. (F) Enrichment score calculated by gene set enrichment analysis using Reactome pathway database (positive enrichment means an enrichment in PT2 cluster). (G) Pathway activity in PT1 and PT2 clusters (inferred from PROGENy). (H) Collagen, extracellular matrix (ecm) proteoglycan (pg) and glycoprotein (gp) scores in PT1 and PT2 clusters. (I) Potential of heat-diffusion for affinity-based trajectory embedding (PHATE) dimension reduction projecting pathway enrichment estimated by GSVA for each cell type. (J and K) Representative immunostainings of HAVCR1 (J) and VCAM-1 (K) proteins in fibrotic area. ∗∗∗p < 0.001 ∗∗∗∗p < 0.0001.

LV025 and LV070 were associated with peculiar cell states identified in the newly generated snRNAseq data from kidney biopsies obtained in the early phase of AKI. In this dataset the PT compartment displayed two main clusters: PT1 was enriched in control samples (and was associated to LV070), whereas PT2 was enriched in AKI samples (and was associated to LV025) (Figures 4A and 4B). PT2 displayed higher levels of injury markers, such as Hepatitis A virus cellular receptor 1 (HAVCR1, also known as KIM1), neutrophil gelatinase-associated lipocalin/lipocalin 2 (LCN2), and insulin-like growth factor binding protein 7 (IGFBP7) and VCAM1 (Figures 4C–4E). The PT2 cluster displayed an enrichment in pathways related to hypoxia, extracellular matrix organization and developmental biology, and a decrease in physiological PT functions, including solute transport and oxidative phosphorylation with fatty acid oxidation (Figure 4F). PROGENY method revealed the induction of TGFβ, NF-κB, TNFα, and WNT pathways (Figure 4G). Focusing on fibrosis, we found higher extracellular matrix scores in PT2 (Figure 4H). A dimensional reduction of the whole-cell types according to their pathway enrichment clustered PT2 cells close to immune and stromal cells (Figure 4I). Moreover, secondary validation by immunohistochemistry consistently showed fibrotic tissue surrounding HAVCR1 and VCAM1 marked cells in the patients with a prolonged delay between AKI onset and kidney biopsy (Figures 4J–4L).

## Discussion

The cellular response to kidney injury has been extensively investigated in the last years, particularly in the PT.^14^ In animal models, single cell technologies allowed a precise characterization of the dynamic PT cell state transitions, starting from the early phase of cell dedifferentiation and death, to the activation of a reparative program including cell proliferation and re-differentiation.^9^^,^^10^ This complex biological process can restore tubular structures and functions, but can also result in a maladaptive response leading to the persistence of altered epithelial cells, which acquire a squamous epithelium morphology and maintain a dedifferentiated cell state. Experimental data suggested that the persistence of altered tubular cells in the kidney is a critical element in the generation and the perpetuation of the *fibrogenic niche*, a microenvironment of chronic inflammation and fibrogenesis surrounding the damage tubules driving secondary and irreversible tissue damage.^11^ The work presented in this article provides evidence that the same mechanisms are relevant in man with a broad applicability in clinical nephrology. Two major elements were required to achieve this aim. First, we generated one of the first single-cell RNAseq datasets covering the first days/weeks after AKI in severely ill patients, a frequent clinical scenario in which it is usually not possible to obtain kidney biopsies. Second, we developed a computational framework to exploit the information obtained by single-cell technologies in small number of patients to analyze clinically relevant patient cohorts.

In the context of the early phase of the COVID19 emergency, the local ethical committee approved our initiative to perform kidney biopsies before planned withdrawal of resuscitation measures in severely ill patients with COVID19-associated AKI. These biopsies were not only important in the clinic for a better understanding of this new clinical entity, but also provided the opportunity to generate a unique snRNAseq dataset to study AKI in humans. The injured PT cell states identified in those patients shared the main features previously characterized in experimental models of ischemic AKI in mice, including the loss of physiological tubule functions and fundamental changes in energy metabolism and the activation of genes related to cell-matrix interaction and tubule development.^8^^,^^10^^,^^14^ Injured PT cells activated the main signaling pathways known to be involved in CKD progression, such as TGFβ, NF-κB, TNFα, and WNT, consistently with their role in the generation of the *niche*.^11^ The observation that the main biological processes of AKI are conserved across species is in line with our previous studies based on bulk RNAseq.^22^ Expanding the granularly of information obtained by single-cell technologies in the clinic is expected to define novel diagnostic and prognostic tools, but also to accelerate the discovery of new drug targets to prevent CKD progression.

The K-CLIER was inspired from the MultiPLIER, a transfer learning approach specifically designed to overcome the limitation of unsupervised machine-learning methods when working with limited number of cases.^16^ Importantly, results have shown that a single K-CLIER model trained on a large public dataset can be effectively exploited in the analysis of other datasets, without any specific tuning. This was particularly important in our area of interest in consideration of the lack of large kidney biopsies dataset analyzed by bulk RNAseq. We adapted the computational framework to use the knowledge obtained from all published studies reporting scRNAseq data from human kidney. The validation process confirmed that the computational framework was extremely powerful to study the kidney by allowing not only a substantial dimensionality reduction without loss of relevant information, but also opening the unique opportunity to link clinical information with single-cell signatures. This approach generated clinically relevant information also in the context of the validation process in kidney cancer, by providing evidence of a link between injured PT cells and papillary renal cell carcinoma (as previously reported)^24^ and on the cellular origin of chromophobe renal cell carcinoma.^20^ The application of the K-CLIER in two rather small cohorts of patients with kidney disease confirmed that the model was able to extract biologically meaningful information. Most importantly, it highlighted the central role of PT cell states in progression of kidney disease and fibrosis. Notably, the computational framework is generally applicable, and its accuracy is expected to improve by expanding the number of signatures to train the system.

### Limitations of the study

Our study has several limitations. First, the single-cell characterization of AKI in our patients might include some COVID19-specific findings.^25^ The atlas of transcriptionally defined cell states should be expanded to include different types of kidney injury and to correctly address the multidimensional complexity of human diseases and possible confounders, such as comorbidities and medication. Moreover, we used the single-cell signatures as reported by the authors of the corresponding publications. An integrated analysis of single cell data to generate the signatures in a more standardized way is likely to improve the accuracy of the computational approach. Second, we decided to focus on PT cells because of their relevance in the unbiased analyses and because this compartment has been particularly well characterized in experimental models, but other cell types are likely to be involved in this process, including endothelial cells and immune cells. As soon as the experimental knowledge on these cell types will be refined, the same strategy could be implemented to study other cell types of interest. Third, the K-CLIER successfully worked on bulk RNAseq from kidney biopsies. Recent studies demonstrated the possibility to perform scRNAseq analyses on urine sample.^26^ This would open the opportunity to recalibrate the system and to develop diagnostic tools with a broader clinical applicability.

In conclusion, we presented an integrated approach to translate the knowledge obtained through the application of single-cell technologies in experimental models of AKI toward clinical nephrology. Our studies provide multi-level evidence for a critical role of altered PT cells in the pathogenesis of CKD.

## STAR★Methods

### Key resources table

**Table:** 

REAGENT or RESOURCE	SOURCE	IDENTIFIER
**Antibodies**		

mouse monoclonal anti-human HAVCR1	clone 219211	MAB1750; RRID: AB_2116559; RD Systems
mouse monoclonal anti-human VCAM1	clone 1.4C3	MA1-12637; RRID: AB_1071130; Invitrogen

**Biological samples**		

Human renal biopsies	University Hospital of Geneve	NA

**Critical commercial assays**		

Chromium Next GEM Chip G Single Cell Kit	10X Genomics	1000120
Chromium Next GEM Single Cell 3' Kit v3.1	10X Genomics	1000268

**Deposited data**		

The Cancer Genome Atlas	The National Cancer Institute’s (NCI’s) Genomic Data Commons (GDC)	https://gdc.cancer.gov/access-data
TPL cohort	GEO	GSE126805
DKD cohort	GEO	GSE142025
snRNAseq control samples	GEO	GSE131882
Raw single nucleus RNAseq	This paper deposited in GEO	GSE185778

**Software and algorithms**		

R version 4	R Development Core Team, 2011	https://www.r-project.org/
CellRanger version 3.1.0	10x Genomics	https://www.10xgenomics.com/support/software/cell-ranger
Seurat v4	Satija Lab	https://satijalab.org/seurat/
ReactomePA version 1.34.0	Bioconductor	Yu et al.^27^
PROGENy version 1	Saez Lab	Holland et al.^28^
GSVA	Bioconductor	Hanzelmann et al.^29^
PLIER	https://github.com/wgmao/PLIER	Mao et al.^15^
SHAP	cran.r-project.org	Mayer et al.^30^^,^^31^
Code	Zenodo	https://zenodo.org/records/8144199?token=eyJhbGciOiJIUzUxMiIsImlhdCI6MTcwOTI5Mjk3OCwiZXhwIjoxNzExODQzMTk5fQ.eyJpZCI6ImMxZmY2NmYxLTEyYzMtNGFhNy1iMGY1LTZhMDNlZTkzN2M2YSIsImRhdGEiOnt9LCJyYW5kb20iOiIzYTI2ODhiY2ZjNGFlZjhiODhmZjkwNDE5YTJiZDRlNiJ9.95CiT0_tBU_d9EIJzn2oRvEO65l2XxxOtICxX1Izt-EJoaeHRipBYfi15C1ZKfcTVR3qwEOAGmxxzP-NRJjF7A

### Resource availability

#### Lead contact

Further information and requests for resources and reagents should be directed to and will be fulfilled by the lead contact, Prof. Pietro Cippà, MD PhD (pietro.cippa@eoc.ch).

#### Materials availability

This study did not generate new unique reagents.

#### Data and code availability

• Raw single nucleus RNA sequencing data generated in this paper have been deposited at GEO and are publicly available as of the date of publication. The public datasets used are from GEO. Accession numbers are listed in the key resources table. With the exception of those specifically derived within this study, all single-cell signatures used to train K-CLIER have already been published and are publicly available (please refer to the corresponding references for further details).
• All original code has been deposited at Zenodo and is publicly available as of the date of publication. DOIs are listed in the key resources table.
• Any additional information required to reanalyze the data reported in this paper is available from the lead contact upon request.

### Experimental model and study participant details

#### COVID-19 associated AKI clinical cohort characterized by single nucleus RNAseq - Study approval

All patients admitted to the intensive care unit of the Geneva University Hospitals between April 5^th^ 2020 and May 15^th^ 2020 were screened. Patients were included if they met the following inclusion criteria: positive COVID19 status defined as a positive PCR for SARS-Cov-2 and pneumonia, older than 18, administration of sedative and class III analgesic and if a decision of treatment withdrawal had been taken by the attending physicians. The consent was given orally by the next of kin, due to the ban on visits in force in our hospital. The study was approved by the local ethical committee for human studies of Geneva, Switzerland (CCER 2020-00644, Commission Cantonale d’Ethique de la Recherche) and performed according to the Declaration of Helsinki principles. Refer to Table 1 for additional details on age, gender, race and clinical information.

### Method details

#### Experimental

##### Kidney biopsies - Single nucleus RNAseq in COVID-19 associated AKI

Once the consent obtained, kidney biopsies were performed by DL and SDS, just before the therapeutic withdrawal. Using 18G Automated biopsy guns (Max-Core, BardCare) in combination with real-time ultrasound guidance. Biopsies were directly frozen and stored at -80°C.

##### Histology and immunochemistry

Human kidney biopsy specimens were fixed with formaldehyde 4%, dehydrated and paraffin-embedded. 2μm kidney sections were stained with hematoxylin and eosin, periodic acid–Schiff, Jones, and Masson's trichrome for evaluation of tubular injury, inflammatory infiltrates and kidney fibrosis. Immunochemistry staining were performed as followed: after antigen retrieval with pressurized heating chamber in citrate buffer pH7 or tris-EDTA pH9, 5μm tissue sections were incubated with antibodies mouse monoclonal anti-human HAVCR1 (dilution 1:250, clone 219211, RD Systems) and mouse monoclonal anti-human VCAM1 (dilution 1:25, clone 1.4C3, Invitrogen) for 1h at room temperature. Then, the slides were incubated with the appropriate horseradish peroxidase–conjugated secondary antibody (Dako, Via Real Carpintera, USA) for 30mn at room temperature. Slides were developed using diaminobenzidine chromogen and then counterstained with Mayer hematoxylin. Stained sections were examined with a Zeiss microscope (Zeiss, Oberkochen, Germany). Negative controls were performed in absence of primary antibody.

##### snRNA-seq sample processing

Single nuclei isolation from tissue was performed as previously described in the genomic facility of the Institute of Oncology Research, Bellinzona, Switzerland.^32^ Briefly, frozen samples were cut into small pieces and transferred into a 2-ml Dounce homogenizer (Sigma, Cat#D8938) loaded with 1 ml of NEZ Lysis Buffer (Sigma, Cat#N3408) with RNase inhibitor (NEB, Cat#M0314) at final concentration of 0.4U/μl on ice. Samples were then Dounce homogenized on ice with five strokes of the looser pestle every 2 min for 8 min (25 strokes in total). Samples were then slowly Dounce homogenized 25 times with the tighter pestle on ice. The homogenized sample was filtered through a 40-μm Falcon Nylon Cell Strainer, then the filter was washed with 8 ml of 1% BSA PBS, and the nuclear suspension spin in a precooled (4°C) centrifuge at 650g for 8 min. Supernatant was removed, the pellet re-suspended in 2% BSA PBS with RNase inhibitor and nuclei from human biopsies further filtered by 20-μm and 10-μm strainers and moved to a low-bind Eppendorf tube. Nuclei from murine samples were sorted as DAPI and EDU positive on a BD Aria into 2% BSA solution with RNA inhibitor after 40um filtering. Nuclear quality and number were assessed with trypan blue staining. Single-cell transcriptomes was performed using 10X Chromium single cell platform (10X Genomics) and processed according to the 10X Chromium protocol. Barcoded single-cell gel beads in emulsion (GEMs) were created by 10x Genomics Chromium TM and then reverse transcribed to generate single-cell RNA-seq libraries using Chromium Single Cell 3’ Library and Gel Bead Kit v2 (10X Genomics) according to manufacturer’s instructions. Resulting short fragment libraries were checked for quality and quantity using an Agilent 2100 Bioanalyzer and Invitrogen Qubit Fluorometer. Sequencing Unique molecular identifiers (UMIs), which were incorporated into the 5’ end of cDNA during reverse transcription, were used to quantify the exact number of transcripts in a cell. Paired-end sequencing was carried out on Illumina NextSeq500platform using 150-cycle High Output.

#### snRNA-seq data analysis

##### snRNA-seq data processing

Sequencing data were processed by CellRanger (version 3.1.0) and reads were aligned to pre-mRNA human reference genome (GRCh38-2020). The Cell Ranger *cellranger count* function output filtered gene–cell expression matrices removing cell barcodes not represented in cells. Finally, a UMI count table utilizing both exonic and intronic reads was generated for downstream analysis. The whole data processing was executed by running the script on the Ente Ospedaliero Cantonale server, Switzerland.

Seurat v4 in R v4 was used for downstream analyses, including normalization, scaling, and clustering of nuclei. First, we analyzed each sample separately and excluded nuclei with less than 150 nFeature_RNA detected or more than 4 times the absolute median of nFeature_RNA. We also excluded nuclei with a relatively high percentage of UMIs mapped to mitochondrial genes (>1%) and ribosomal genes (>1%). Subsequently, we applied SoupX to remove ambient RNA contamination from the human samples. Ambient RNA was estimated from the empty droplet pool with setting “nonExpressedGeneList” to hemoglobin genes (https://github.com/constantAmateur/SoupX). We performed curated doublet removal based on known lineage-specific markers. The samples were integrated to avoid batch effect using Seurat standard work flow split by sample (*orig.ident)*. Following ScaleData, RunPCA, FindNeighbours and FindCluster at a resolution of 0.5 were performed. FindAllMarkers generated the list of genes differentially expressed in each cluster compared to all other cells, within the major subgroups defined (nephron cells, collecting duct, other cells) based on the Wilcoxon rank-sum test and limiting the analysis to upregulated genes with a cut-off for minimum log fold change difference 0.25) and minimum cells with expression (0.1). Cluster reassignment was performed based on manual review of lineage-specific marker expression.

Feature plots were drawn as scatterplots from a given reduction showing the gene-weighted density, using the Nebulosa package.

Secondary Seurat analyses on PT cells employed the SubsetData function to create new r-objects from cohorts of primary analysis. On the subset object, we applied the RunPCA function with default parameters and RunUMAP on 30 dimensions with n.neighbors and min.dist set to 50 and 0.01 respectively.

##### Pathway analyses

General pathway enrichment score between PT1 and PT2 cells was performed with the gsePathway function from the ReactomePA^27^ (v1.34.0) package, using a false discovery rate of 0.1 and a minimal geneSet size of 1. The input list of genes was extracted from the Seurat object, with the FindAllMarkers function, using a Wilcoxon test) and sorting according to the log-fold-change variable.

The collagen, extracellular matrix, proteoglycan and glycoprotein scores in PT1 and PT2 was calculated as previously described.^4^

Progeny (v1)^28^^,^^33^ was used to analyze activities of key biological pathways in individual cells. Briefly, gene weights for each pathway were defined which considered top 1000 significant genes per pathway. Then, the dataset was normalized and scaled, and 100 permutations were conducted to calculate p-values of random activities.

GSVA was performed on all cells. We further applied the Potential of Heat diffusion for Affinity-based Transition Embedding method^34^ with default parameters to project each cell type in a two-dimensions scatter plot.

##### Pseudobulk analyses

To identify cell type-specific transcriptional effects of AKI, we performed differential gene expression on “pseudo-bulk” expression profiles.^35^ We firstly generated pseudo-bulk matrix by summing counts together for all cells with the same combination of label and sample. We thus used quasi-likelihood (QL) methods from the edgeR package to perform differential gene expression.

#### Public datasets

This section provides a description of the previously published data resources used in our work, which include publicly available datasets and single-cell signatures extracted from the existing literature.

##### Data for developing diagnostic and prognostic machine learning models

To train K-CLIER and develop machine learning models, we employed three datasets. Here, we provide descriptions of these datasets with a focus on the information that is relevant for their use in machine learning.

###### TCGA

The Cancer Genome Atlas (TCGA) is a program aiming at creating a large publicly available database of oncological data. We used a gene expression dataset created from biopsies of patients affected by 33 different types of cancer (11.348 samples in total). We downloaded TCGA’s genetic expression data in TPM through the Bioconductor package *recount3*.^36^ The TPM data were used to train K-CLIER. In addition to TPM data, we downloaded from the project’s website demographic, clinical and laboratory variables. Some of the analyses in our work focus on two specific subsets within TCGA. The first is the kidney sub-dataset, hereafter referred to as KIPAN (Pan-kidney cohort, comprising 1,026 samples); the second is a more specific sub-dataset within KIPAN, consisting of samples associated with clear cell carcinoma (KIRC) tumors (531 samples). The variables that serve as targets for machine learning model predictions include, for the KIPAN dataset, the tumor histological type (531 KIRC samples, 290 KIRP samples, 66 KICH samples, and 139 Normal samples), as well as, for the KIRC sub-dataset, the binary Overall Survival (357 records labeled as 0, 174 labeled as 1) and the associated time for Overall Survival.

###### DKD cohort

The dataset contains gene expression data from biopsies of 37 patients affected by diabetic nephropathy (6 with early nephropathy, 22 with advanced nephropathy) and control patients (9).^21^ We downloaded raw counts data (GSE142025) from the Gene Expression Omnibus (GEO) and processed them using the same pipeline as *recount3*. The nephropathy diagnosis is based on histological evidence, and the advanced state of nephropathy is used as a binary target for machine learning models.

###### TPL cohort

The dataset contains gene expression data from biopsies of 42 patients subjected to kidney transplant.^22^ For each patient four biopsies at different time points are present: before reperfusion, after reperfusion, 3 and 12 months after transplant. The dataset is publicly (GSE126805) available and we downloaded genetic expression data in TPM through *recount3*. In addition to gene expression data, the dataset contains several clinical variables. Notably, it includes the ci-score (Banff classification) derived from biopsies at 12 months after transplant, which we converted into a binary variable (ci-score>1). This binary variable served as the target for our machine learning models. It is important to note that in the manuscript we refer to the ensemble of biopsies extracted at 12 months after transplantation as the "TPL" cohort (32 samples, 12 with ci-score>1), while we use "TPL-P" for the ones extracted at 3 months (30 samples, 9 with ci-score >1). The TPL cohort is used for diagnostic machine learning models, whereas the TPL-P cohort for prognostic models.

#### Public single nucleus RNA-Seq dataset integrated

##### snRNAseq controls

The dataset was downloaded from Gene Expression Omnibus (http://www.ncbi.nlm.nih.gov/geo/) (GSE131882) and comprises 3 early human diabetic kidney samples and 3 controls characterized by single nucleus RNA sequencing.^37^ Control samples represent non-tumor tissue in patients undergoing nephrectomy for renal mass. Only the control samples were integrated to the COVID-19 dataset.

#### Kidney-specific single-cell signatures atlas

The atlas includes all renal single-cell signatures reported in the literature in studies focused on the human kidney published from August 2018 to May 2021,^4^^,^^6^^,^^17^^,^^19^^,^^37^^,^^38^^,^^39^^,^^40^^,^^41^^,^^42^^,^^43^^,^^44^^,^^45^^,^^46^^,^^47^^,^^48^ and additionally 19 new signatures of acute kidney injury generated in this study (Figure 1B; Table S1), that we describe in detail in the following section. The atlas reflects all cellular compartments of the kidney (Cell Type category) in non-pathological and diseased kidneys. Based on the clinical classification, it includes signatures obtained from: (i) non-pathological adult (n=142) and fetal (n=56) kidneys, (ii) diseased kidneys, including cancer (n=294), diabetic nephropathy (n=26), lupus nephrites (n=9), acute (n=19) and chronic kidney disease (n=137), transplant (n=31), (iii) organoids (n=3) and (iv) urine (n=8) specimens. The label of the signature includes the name of the first author of the original study, indication of the species (h, Homo sapiens), clinical classification and cell type. Signatures that were not associated to a specific cell type were classified as “Non associated” (n=66).

### Quantification and statistical analysis

In this section, we provide insights into the PLIER algorithm and how it was employed in training K-CLIER (by using the TCGA dataset and the aforementioned signatures atlas). Then we briefly present GSVA, which is another possible approach to genetic data. Lastly, we present statistical methods, machine learning models, and data analysis tools that we used both to assess K-CLIER’s effectiveness and to address the main study questions of our work. Machine learning models are described using the checklist proposed by Luo et al.^49^ as a reference point. Additionally, we assessed the models using the PROBAST approach outlined in,^50^ and we are confident that they all exhibit a low risk of bias.

#### PLIER algorithm

The PLIER algorithm, introduced by Mao et al. in 2019,^15^ is a feature extraction method that enables the representation of high dimensional gene expression matrices using a lower number of variables, some of which are interpretable, without relevant loss of information. Given an N×M gene expression matrix Y, with M the number of samples and N the number of genes, PLIER decomposes it as the product Y≈ZB, where Z is an N×K matrix of “loadings” and B is a K×M matrix of “scores”. The K rows of B are analogous to the principal components of a traditional singular value decomposition (SVD), but, following Mao’s paper, hereafter will be referred to as latent variables (LVs) to stress the fact that they are not orthogonal. In fact, in the PLIER decomposition orthogonality is lost when encouraging Z to align with prior knowledge. This is achieved by defining an N×S prior knowledge matrix C, where each column represents a gene signature, assigning 1 to the genes included in the signatures and 0 to the other genes. The matrix Z is then encouraged to satisfy Z≈CU, that is, to resemble the product of C with an S×K matrix of signature scores called U. The desired result is attained by minimizing a loss function that includes the Frobenius distance between Y and ZB, and between Z and CU, along with penalty terms. Specifically, matrix B is penalized with L2 penalty, and matrix U with L1 penalty, where the latter ensures that each LV is represented by only a few signatures. The decomposition is typically learned on a large gene expression dataset, and is then applied to a new dataset. For any new gene expression dataset Y’, the matrix B’ is calculated, using the Z matrix learned in the training. B’ contains the LVs scores for the new dataset that can be used for further analysis. It's worth noting that LV scores are generally less correlated than gene expression variables, which tend to exhibit high correlation patterns.

Mao et al. 2019 proposed a method to measure to what extent a non-zero weight in the U matrix represents a real association between a LV and a signature. This involves a new run of PLIER where a random selection of one-fifth of the genes from each signature in the matrix C is set to zero. By comparing the loadings (in the Z matrix) assigned to the left-out genes to those assigned to the other genes not belonging to the signature, for each LV-signature pair with a positive score in the U matrix, the method produces an AUC and an associated p-value of the Mann-Whitney test. The AUC measures the probability that a left-out gene is assigned a bigger loading than a gene not belonging to the signature, whereas the p-value measures how significantly higher than 0.5 such probability is. P-values are then corrected to obtain false discovery rates (FDRs). The validity of such measures of association between LVs and signatures was demonstrated by the authors of Mao et al. (2019) in several experiments, showing how well LVs strongly associated with specific signatures are correlated with associated biological observations (for instance it was shown that LVs strongly associated with cell-type markers are correlated also with the corresponding cell proportions). As a criterion for association, the authors proposed satisfying both AUC>0.7 and FDR<0.05.

#### K-CLIER transformation

We used the PLIER algorithm to learn our Kidney-specific Cell-level Information ExtractoR, or the K-CLIER transformation. We fed PLIER with the atlas of kidney-specific single-cell signatures, as prior knowledge, and the TCGA dataset (see section “Datasets” for a description), as gene expression dataset.

For TCGA, we considered the TPM measures of all the samples (n=11.348). We removed genes for which the expression across all samples had null variance or more than 95% of values equal to zero. Genes not included in any signature were also excluded from the analyses. This resulted in 18.438 genes included in the model. Due to strong skewness of TPM data, we used as gene expression measure a logarithmic transformation of the TPM, i.e., log2(TPM+0.5).

The training was conducted using the *plier* function in the *PLIER* R package. We set the parameter defining the number of latent variables to k=700. This number was selected using the permutation methods proposed by Mao et al. 2019 and implemented in the *num.pc* function of the package. We verified that, as pointed out in Mao et al. 2019, the method is not sensitive to the exact value of k by comparing the results obtained with different choices of k. We set the parameter *frac* to 0.5 (and not to the default value 0.7), to increase the sparsity of the U matrix, which is desirable for our interpretation purposes. We performed a few experiments with different choices of *frac* as well, and we encountered stability and consistency in the results. For the remaining parameters of the *plier* function, including the three free parameters describing the relative weights of the loss function terms, we used the default values.

During the training phase, we learned the Z and U matrices and we computed the AUC and FDR values measuring the strength of association between each LV-signature pair having a non-null score in the U matrix. Following the criteria of association^26^ proposed by Mao et al., i.e., AUC>0.7 and FDR<0.05, 96 out of 700 LVs in the model were strongly related to at least one signature.

To analyze the relevance of a LV-signature association to the biological system under study, the AUC and FDR should be considered together with the U matrix scores. In fact, while the AUC and FDR measure the strength of the association between a LV and the relative signatures (high AUC and low FDR implying a strong association), the U matrix scores specify to what extent an increase in the LV expression will correspond to an increase in the cell type proportions: indeed, if the U matrix weight is low, regardless of the possibly strong correlation between the LV and the cell type, one can expect only small variation of the cell type proportion when the LV varies.

K-CLIER can be applied to any new TPM gene expression dataset that has undergone the same preprocessing as the training one. In this way, for each sample, we obtain the LVs scores to be used for further analyses. At first, we tested the transformation in-sample, by applying it directly to TCGA itself (this is possible since K-CLIER performs an unsupervised decomposition). We then tested the transformation out-of-sample, by applying it to a few other datasets (i.e., DKD, TPL, and TPL-P cohorts).

#### Gene set variation analysis (GSVA)

To assess the merits of K-CLIER, we compared it to a reference method, i.e., gene set enrichment analysis based on GSVA.^29^ In contrast to the PLIER transformation, which assigns a score vector to each sample (represented by the columns of the B matrix) to summarize the information conveyed by gene counts and establish a connection with specific signatures of interest, gene set enrichment analysis provides a more direct and conventional approach to condense gene expression information into a summary measure related to signatures. This approach lacks the training stage in which relevant signatures are combined or selected, which is performed by the PLIER when learning the U matrix. However, it can still be used to perform all the analysis presented below, e.g., it can provide scores of signature enrichment that play the same role as the LVs scores. We applied GSVA to TCGA genetic data to obtain the enrichment scores for each single-cell signature in the kidney-specific single-cell signatures atlas. We used the method implemented in the function gsva of the Bioconductor package GSVA.

We compared correlation patterns of variables produced by applying GSVA to TCGA, with those from gene expression variables and those from LVs and, in the KIRC dataset, we also compared the use of GSVA variables and LVs for machine learning purposes (Figure S1). For both sets of variables we trained prediction models for overall survival and we performed multivariate survival analysis.

#### Multiple tests

To evaluate the differential expression of a LV between two sub-populations of patients, we performed a Mann-Whitney test, which is a non-parametric statistical test to assess whether one of the two samples is drawn from a population that is stochastically greater than the other. Since the test was performed on a large number of LVs, we adjusted the p-values for multiple tests by means of the Benjamini-Hochberg correction to control the false discovery rate.

We employed Mann-Whitney test (in its unpaired version) to assess differences between two samples in several different contexts: patients with different histological types (KIRC dataset), patients affected or not by a disease (DKD, TPL and TPL-P datasets). In all cases two-sided tests were performed, since *a priori* there was no reason to test “upregulation” or “downregulation” for a given LV.

#### Classification models

We developed a number of multivariate classification models, leveraging various datasets, and frequently employing several different variables sets for each dataset. As a general observation applicable to all models, none of the variables had any missing values, eliminating the need for any imputation procedures. Furthermore, we did not carry out any outlier detection procedures.

For the KIPAN dataset, we developed diagnostic models aimed at classifying histological types. This set of models includes two models designed to estimate prediction performance and one created solely for interpretation purposes. All of these models were random forest models, trained using the *randomForest* function from the homonymous R package,^51^ with default parameters. For the performance estimation models, we considered two scenarios: one using all 700 available LVs, and one using exclusively the interpretable subset of LVs (a total of 96). In both cases, we conducted 10 repetitions of a stratified 10-fold cross-validation, and then pooled together predictions across different folds within the same repetition. We refrained from performing any hyperparameter tuning, as the default parameter values consistently yielded nearly perfect classifiers (AUROC > 0.99) for all repetitions. We also trained a model on a single repetition on the entire dataset, using only the 96 interpretable LVs, to provide a basis for feature importance analyses using SHAP (Figure 2D). Since the analogous cross-validation model achieved almost perfect classification, we opted not to perform any hyperparameter tuning for this model as well.

We trained prognostic models to predict the binary Overall Survival of patients in the KIRC dataset (see Figure S1). These models were trained using various sets of variables, including gene expression variables (18,630), GSVA variables (725), LVs (700), interpretable LVs (96), clinical variables (5), and a combination of LVs and clinical variables (705 in total). We used a logistic regression model with lasso penalty, in order to perform automatic feature selection, which was especially necessary for the gene model due to the large number of variables. The models were trained using a stratified 10-fold cross-validation repeated for 10 times, and the choice of the penalization parameter was performed by means of the function cv.glmnet using a nested 10-fold cross-validation. The models were implemented by means of the glmnet R package.^52^

We developed several distinct models aimed at predicting Chronic Kidney Disease (CKD) in various contexts, namely diagnostic models (for the DKD and TPL cohorts) and prognostic models (for the TPL-P cohort). For each cohort, we trained six different models, each employing distinct groups of LVs associated with different cell types (Endothelial, Proximal Tubules, Stroma, Myeloid, Lymphoid). For the Endothelial case, the datasets featured only two LVs, whereas for the other cases they included four. All models underwent stratified 10-fold cross-validation repeated for 10 times, and predictions were subsequently aggregated across different folds within the same repetition. The results of these analyses can be found in Figure 3B and in the upper portion of Figure 3C, which focuses on Proximal Tubules. For each cohort, we also trained an extra model on a single repetition on the entire dataset using Proximal Tubules variables, primarily for interpretation purposes with SHAP (lower portion of Figure 3C). For all models predicting CKD, we used the *randomForest* function from the R package^32^ with default parameters. We refrained from hyperparameter tuning due to the small size of the datasets, that would not permit us to conduct the tuning in a nested cross-validation setting. Note that the use of a repeated cross-validation is particularly relevant for small datasets, as in such cases the division into folds could have a non-negligible impact on the performance metrics. In addition to random forest models, we also trained logistic regression models. Since these yielded conclusions similar to the random forest ones, we chose to omit them from the discussion and figures for the sake of simplicity.

The performances of the classification models were assessed in terms of area under the receiver operating characteristic curve (AUROC) and in some cases of area under the precision recall curve (AUPR). The AUROC can be considered a reliable performance metric for all models, as none of the training scenarios presented a severe class imbalance.

#### Survival analysis

The multivariate survival analysis in Figure S1, conducted on the KIRC dataset, was performed by means of a Cox model with lasso penalization, in order to select the most relevant features. The different sets of variables used for the models are the same as for the classification models for Overall Survival. The training was also performed in the same setting as for classification models, namely by means of a stratified 10-fold cross-validation repeated for 10 times, with pooling of predictions within the same repetition. The Cox model was implemented by means of the *glmnet* R package. The choice of the penalization parameter was performed by means of the function *cv.glmnet*. The performance of the model was assessed in terms of concordance c-index.

#### UMAP

To perform dimensionality reduction for data visualization, we used the Uniform Manifold Approximation and Projection, UMAP. This is a non-linear dimensionality reduction method based on manifold learning techniques. UMAP was implemented by means of the *umap* R package^53^ with default parameters.

#### SHAP

For model interpretation, we used SHapley Additive ExPlanations (SHAP).^54^ This is a method based on Shapley values from game theory. In general, for each variable and instance we calculated the SHAP value. We defined features importance by ranking the features by decreasing mean absolute value of the SHAP values.

Computation and visualization of SHAP values was performed by means of the R packages *kernelshap*^30^ and *shapviz*.^31^ For the histological type prediction model, the generation of each subfigure required a few hours of computation. A tree-specific explainer would have been faster, but at the moment (to the best of our knowledge) no R package provides it for multiclass random forest.

## Supplemental information

Document S1. Figure S1

Table S1. List of the single-cell signatures included in the ATLAS, related to STAR Methods and Figure 1

Table S2. Matrix of association between LV and single-cell signatures, related to Figure 2
