# Supplementary material for: A transfer learning framework to elucidate the clinical relevance of altered proximal tubule cell states in kidney disease
Source: iScience. 2024 Feb 22;27(3):109271. doi: 10.1016/j.isci.2024.109271 (PMC10937833; doi:10.1016/j.isci.2024.109271)
Supplement: Document S1. Figure S1 [file mmc1.pdf]

iScience, Volume *27*

Supplemental information

A transfer learning framework to elucidate the clinical relevance of altered proximal tubule cell states in kidney disease

David Legouis, Anna Rinaldi, Daniele Malpetti, Gregoire Arnoux, Thomas Verissimo, Anna Faivre, Francesca Mangili, Andrea Rinaldi, Lorenzo Ruinelli, Jerome Pugin, Solange Moll, Luca Clivio, Marco Bolis, Sophie de Seigneux, Laura Azzimonti, and Pietro E. Cippà

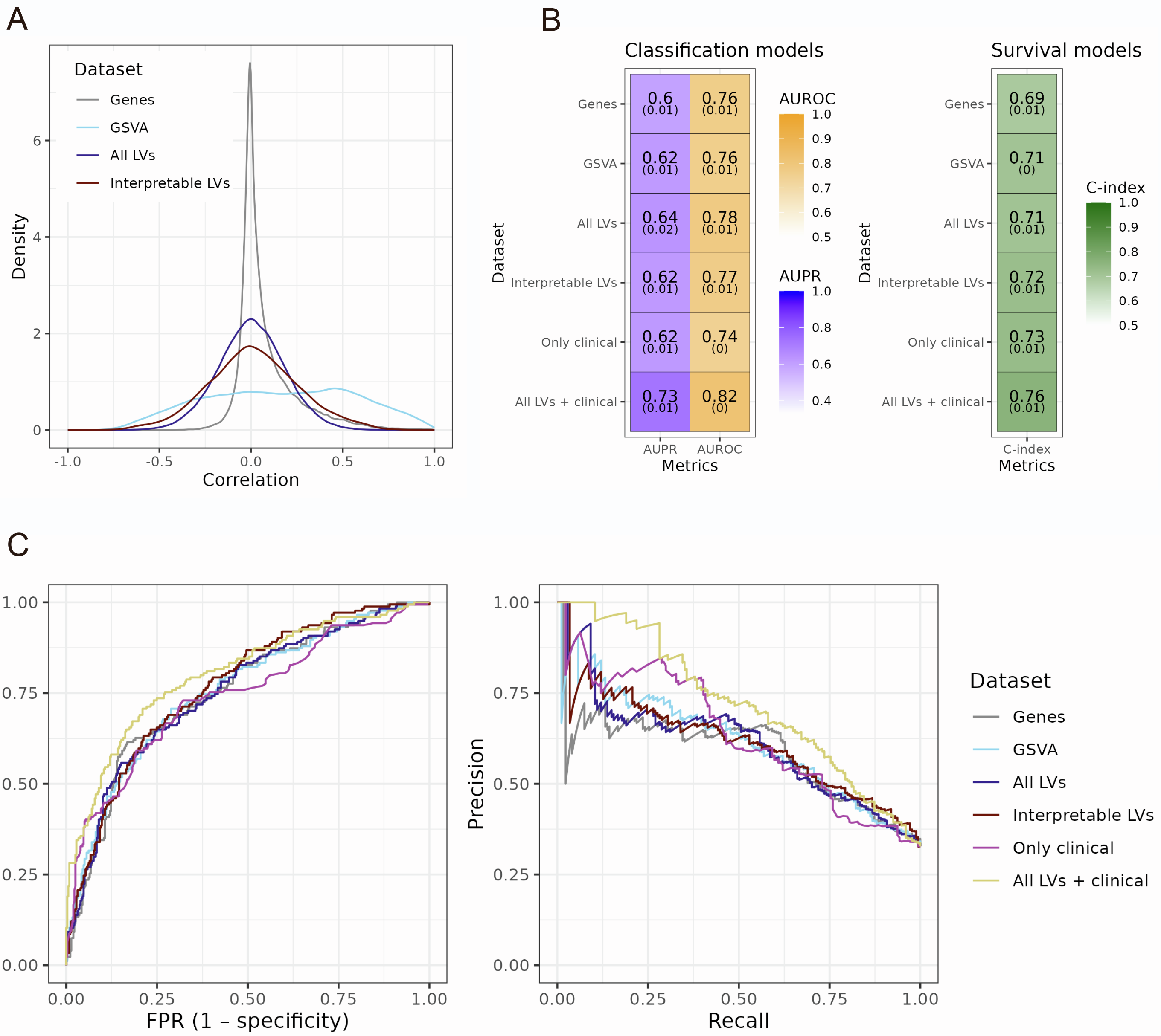

Supplementary Fig. 1. Genes, GSVA variables and LVs, Related to Figure 2. A, Correlation distributions for three types of variables: original genes expression variables (TPM), variables obtained through GSVA, all LVs obtained using K-CLIER, and the subset of interpretable LVs. For genes, a random sample of one million different variable pairs was considered due to the large number of variables. In contrast, correlations between all possible pairs of variables were computed for GSVA variables and LVs. LVs generally exhibit lower correlations compared to GSVA variables. **B,** Performances of classification and survival models in predicting Overall Survival for TCGA KIPAN patients of histologic type KIRC. Multiple datasets were employed, consisting of gene expression variables (TPM), GSVA variables, all LVs, interpretable LVs, clinical variables (metastasis, stage, white cells count, serum calcium), and a combined dataset that included all LVs and clinical variables. For each dataset, 10 repetitions in cross-validation were realized (using different divisions into folds). The left panel shows performances (mean and standard deviation of AUROC and AUPR across repetitions) for a logistic regression model with lasso regularization; the right panel shows performances for a Cox model (mean and standard deviation of C-index across repetitions). In both cases, models trained on genes, GSVA variables, and LVs show comparable performances. Models trained using both LVs and clinical variables show better performances than models using only one of the two groups of variables, thus suggesting that LVs and clinical variables provide complementary information. **C,** ROC and PR curves for the 10th repetition of the classification models in **B**.
